# CD117/c-KIT Expression in Phyllodes Tumor of the Breast and Its Correlation With Morphology and Clinical Outcome

**DOI:** 10.7759/cureus.14914

**Published:** 2021-05-09

**Authors:** Sidra Jahangir, Asif Loya, Sajid Mushtaq, Noreen Akhter, Atif A Hashmi

**Affiliations:** 1 Pathology, Shaukat Khanum Memorial Cancer Hospital and Research Centre, Lahore, PAK; 2 Pathology, Dallah Hospital, Riyadh, SAU; 3 Pathology, Liaquat National Hospital and Medical College, Karachi, PAK

**Keywords:** cd 117 expression, phyllodes tumor, prognostic assessment, c-kit, recurrence

## Abstract

Introduction

Breast phyllodes tumor’s (PT) clinical behavior is difficult to predict on histology. There is limited amount of data available on the role of biological markers. The objective of this study was to assess the expression of CD117 (c-KIT) in PT of the breast and its relationship with morphology and clinical outcome.

Methods

A total of 78 patients having available clinical records between 2004 and 2014 with breast PT were retrieved from the cancer registry at Shaukat Khanum Memorial Cancer Hospital and Research Centre, Lahore and were followed up. Immunohistochemical studies were performed on all the cases using mono­clonal antibody CD117 (DAKO A4502) using Leica Bond-Max automated biosystem (Leica Biosystems, Melbourne, Australia). Staining proportion and intensity of both epithelial and stromal elements were analyzed. Evaluation of the protein expression was determined and scored.

Results

Patients’ mean age was 45.13 ± 11.4 years. Thirty-nine (50.0%) patients had tumor on right side, 38 (48.7%) had on left and only one (1.3%) was bilateral. Thirty-two (41.0%) patients had benign PT, 21 (26.9%) had borderline and 25 (32.1%) had malignant PT. Negative CD117 expression was observed in 20 (62.5%), eight (38.0%) and 12 (48.0%) of benign, borderline and malignant PTs, respectively. Positive CD117 expression was observed in 12 (37.5%) benign, 13 (61.9%) borderline and 13 (52.0%) malignant PTs. There was insignificant association between CD117 expression with respect to tumor sub-type, patient’s age and clinical behavior (p-value > 0.05).

Conclusion

CD117 expression was not found to be associated with tumor type and was not associated with increased risk of recurrence in this sample of patients. Further work is needed to better understand the prognostic value of CD117 expression in breast PTs.

## Introduction

The phyllodes tumors (PTs) are the rare breast lesions having unpredictable behavior that is difficult to determine [1]. These infrequent fibroepithelial neoplasms account for 1% of all breast neoplasms [2]. Initially, Muller was the first to describe PTs in 1838 as cystosarcoma phyllodes [3]. Histologically, PTs have been categorized as benign, borderline, and malignant as per World Health Organization (WHO) criteria [4].

Among PTs the most frequent are benign (64%), followed by borderline (40%) and malignant PTs reach up to 30% [5]. The initial assessment to evaluate PTs involves triple assessment which consists of clinical, radiological and histological examination [6]. Traditionally, the histological grade, stromal overgrowth, tumor necrosis have been used as predictors of clinical outcome [7]. The classification and grading of PTs into benign, borderline and malignant is based on the histological characteristics including degree of stromal hypercellularity, stromal cytological atypia and mitotic activity, stromal overgrowth, and circumscribed vs. permeative margins [8]. A number of studies conducted reported that the adequacy of surgical margins is more important and that histologic features have an inconsistent predictive role [9].

A study conducted by Spitaleri et al. reported around 172 women with PTs, and reported good prognosis for PTs [10]. Another study conducted at Singapore hospital reported 259 cases of breast PTs and concluded that increase risk of recurrence was associated with number of mitosis, surgical margin status and stromal overgrowth [11].

CD117 is a proto-oncogene that encodes a tyrosine kinase receptor [12]. Its expression in mammary PT has not been widely examined, with the stated case numbers being less than 50 [13]. Recently, CD117 expression was also observed in other tumors, while its influence on recurrence was not clearly addressed and still debatable [14,15]. In this study, we employed CD117 immunohistochemistry on PTs (including benign, borderline and malignant PTs) and correlate the results with histological parameters and clinical outcome.

## Materials and methods

Case selection

The study was performed after taking the ethical approval from Shaukat Khanum Hospital and Research Center research board. The analytical cross-sectional study was conducted including 78 patients having clinical record of breast PTs between 2004 and 2014 and the cases with the original diagnosis of PT. Only excision specimens were included in the study. The specimens included were simple mastectomies (for larger tumors) and lumpectomies (for smaller) tumors. All specimens were received in 10% buffered formalin. Gross examination of the specimens was conducted according to standard protocols, as described in previous studies [16-20]. The representative sections were taken and submitted from tumor including interface with normal breast tissue and surgical excision margins. All inadequate biopsies were excluded from the study because of disagreement. The data were retrieved from the cancer registry at Shaukat Khanum Memorial Cancer Hospital and Research Centre, Lahore and were followed up. Assessment of the protein expression was determined and scored. No samples suggestive of other histological findings were included in this study.

Tissue collection

The PT diagnosed and paraffin-embedded tissues of 78 cases of breast over the decade were retrieved. The 4-μm sections from the paraffin-embedded tissue was cut using microtome and stained with hematoxylin and eosin (H&E) [21]. The 78 cases selected were classified into three categories: benign, borderline, and malignant PTs based on the World Health Organization (WHO) criteria including stromal cellularity, stromal atypia, stromal overgrowth, mitotic count, tumor border, and malignant heterologous elements.

Immunohistochemistry for CD117

Four micrometer thick sections were cut on charged slides and air dried overnight. BondMax (Leica Biosystems, Melbourne, Australia) system was used for immunohistochemistry (IHC). Concentrated polyclonal CD117 primary antibody (DAKO A4502) with 1:400 dilution was used. Tissue section was pretreated with Bond Epitope Retrieval Solution 2 (Leica AR9640) for 30 minutes and then incubated with CD117 primary antibody for 30 minutes at 37°C. Bond Polymer Refine Detection kit (Leica DS9800) was used for the detection. External and internal quality controls were used for staining quality evaluation.

The two independent histopathologists reviewed all stained slides. In each slide, high-power microscopic fields (40x magnification) were used for evaluation.

Grading of CD117 immunohistochemical expression

**Figure 1:**
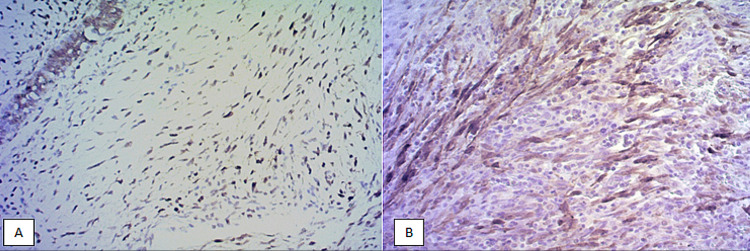
CD117 expression according to tumor type. (A) Microphotograph of benign phyllodes tumor with mild expression of CD117. (B) Microphotograph of malignant phyllodes tumor with strong expression of CD117.

The neoplastic stromal tumor cells show a brown staining for the CD117 expression whether the staining was localized to the cytoplasm or cell membrane or both cytoplasmic and membranous. The results were recorded positive when they showed cytoplasmic or membranous or both staining ranging from moderate to strong. By consensus among the pathologists, the intensity of the immunoreactivity cells was scored as follows; Grade 0: 0% of neoplastic stromal cells positive, Grade 1: 1% to 25% of neoplastic stromal cells positive, Grade 2: 26% to 50% of neoplastic stromal cells positive and Grade 3: 51% or more neoplastic stromal cells positive. Samples with grades 0 and 1 were considered negative. Samples with grades 2 and 3 were considered positives [6,7]. Microscopy of the slides was done and photographed at 200× magnification utilizing a digital microscope camera (Olympus AX80 DP21; Olympus, Tokyo, Japan) interfaced with a computer (Figure 1A, 1B).

Statistical analysis

Data were entered and analyzed by Statistical Package for Social Sciences, version 26.0 (IBM Corp., Armonk, NY). All the quantitative variables were presented in Mean+SD and qualitative variables in frequency and percentages. Chi-square test was applied to check the association between CD117 expression and tumor type and clinical behavior. Independent t-test was applied to check the association between age and CD117 expression. P-values < 0.05 were considered as significant.

## Results

The mean age of patients was 45.13+11.4 years. Thirty-nine (50.0%) patients had tumor on right side, 38 (48.7%) had on left and only one (1.3%) had bilateral disease. Thirty-two (41.0%) patients had benign, 21 (26.9%) had borderline and 25 (32.1%) had malignant PT (Table 1).

**Table 1:** Clinical characteristics of the patients included in the study SD: Standard deviation

Variables	Frequency (%)
Age (Mean±SD)	45.13±11.4
Laterality	
Left	38 (48.7)
Right	39 (50.0)
Bilateral	1 (1.3)
Tumor type	
Benign	32 (41.0)
Borderline	21 (26.9)
Malignant	25 (32.1)

Negative CD117 expression was observed in benign (20, 62.5%), borderline (8, 38.0%) and malignant (12, 48.0%) PTs. Positive CD117 expression was observed in benign (12, 37.5%), borderline (13, 61.9%) and malignant (13, 52.0%) PTs.

There was insignificant association between CD117 expression with respect to tumor type and clinical behavior (p-value > 0.05) and insignificant association between age and CD117 expression (P-value > 0.05) (Table 2).

**Table 2:** Association of CD117 expression with tumor type and clinical behavior SD: Standard deviation

Variables	Frequency (%)		Total	P-value
	CD117 expression			
	Negative	Positive		
Age (Mean±SD)	45±10.0	46±12	45.13±11.4	0.38
Tumor type				
Benign	20	12	32	0.204
Borderline	8	13	21	
Malignant	12	13	25	
Total	40	38	78	
Clinical behavior				
No recurrence	34	26	60	0.082
Recurrence	6	12	18	
Total	40	38	78	

## Discussion

PTs are rare fibroepithelial tumors of the breast that can present as benign, borderline or malignant neoplasms. PTs were usually known as primary stromal lesions, which further show overgrowth and forms tumor. Clinically these tumors sometime behave with uncertainty, as no established predictive markers available for PTs. Stromal cell cellularity, atypia, margin status and pleomorphism are the best prognostic parameters of tumor behavior. In PTs, malignant lesions comprise of 5%-30% [22,23].

In the current study, the mean age of patients was 45.13+11.4 years. Different studies reported different ages for the development of PTs. A study conducted by Tsang et al. reported that mean age of patients was 43 years [24]. In a study conducted in Turkey, 30 females with PTs had median age of 26 years [25]. Another study conducted on malignant PTs of breast and its association between race and clinical features reported that the mean age of patients was 51.7 years and Blacks were younger than Whites (45.7 vs. 55.1) [26]. In another study, the overall patient age range was 14-77 years with mean age of 42 years [27].

In our study, majority of the cases were benign and borderline tumors. CD117 protein expression within stromal cell was insignificantly associated with tumor type and recurrence of disease. It can be observed that only 13 malignant cases showed CD117 stromal positivity, which is the reason of insignificant association. Although in some studies it was reported that there was significant association between grades of tumor and CD117 protein expression. It was also reported that malignant expression of PTs was associated with CD117 [28]. It was also observed that strong expression of c-KIT was found in 50% of malignant tumors and 5% of benign tumors [29].

Increased c-KIT (CD117 expression) in PTs showed positive rate in 29% benign, 17% borderline and 24% frank malignant tumors. Degree of CD117 expression increases as degree of malignancy increases [30]. In contrast to this, another study reported that they did not find any significant relationship of c-KIT expression in benign and malignant lesions [31].

In our study, 60 patients have no recurrence and 18 have recurrent disease. There was insignificant association between clinical behavior and CD117 expression (p-value > 0.05).

Our study is limited owing to small sample size and single-institution data. According to published literature, studies have shown that CD117 expression increased from benign to malignant tumors but few studies deny that, so there is need of new research in this aspect to establish strong relationship between CD117 and PT of breast.

## Conclusions

PTs of the breast are second most common stromal breast lesions after fibroadenoma and their clinical behavior ranges from benign to malignant disease course. Unfortunately, unlike breast carcinoma, there is only limited availability of biomarkers to predict clinical behavior in PTs. In current study, we found insignificant association between PTs of breast and CD117 expression and no association observed with CD117 expression with clinical recurrence of disease. Further studies are required on larger scale to better understand the prognostic value of CD117 expression in PTs of the breast.

## References

[1] Nguyen NT, Maciolek LM, Qiu S, Sadruddin S, Nguyen QD (2020). Malignant phyllodes tumor of the breast in a 26-year-old woman. Cureus.

[2] Grujic D, Cristian H, Hoinoiu T, Miclauș CD, Cerbu S, Grujic L, Oprean C (2021). Skin-reducing mastectomy and immediate reconstruction for a large recurrent borderline phyllodes tumor. Appl Sci.

[3] Abdel Azim H, Abdel-Rahman O, Abdel-Malek R (2017). Bilateral phyllodes tumor of the breast; a case report of benign tumor on one side and malignant tumor on the contralateral side. Res Oncol.

[4] Sevinç Aİ, Aksoy SÖ, Güray Durak M, Balcı P (2018). Is the extent of surgical resection important in patient outcome in benign and borderline phyllodes tumors of the breast?. Turk J Med Sci.

[5] Tan BY, Tan PH (2018). A diagnostic approach to fibroepithelial breast lesions. Surg Pathol Clin.

[6] Toussaint A, Piaget-Rossel R, Stormacq C, Mathevet P, Lepigeon K, Taffé P (2021). Width of margins in phyllodes tumors of the breast: the controversy drags on?-a systematic review and meta-analysis. Breast Cancer Res Treat.

[7] Mihai R, Callagy G, Qassid OL (2021). Correlations of morphological features and surgical management with clinical outcome in a multicentre study of 241 phyllodes tumours of the breast. Histopathology.

[8] Lu Y, Chen Y, Zhu L, Cartwright P, Song E, Jacobs L, Chen K (2019). Local recurrence of benign, borderline, and malignant phyllodes tumors of the breast: a systematic review and meta-analysis. Ann Surg Oncol.

[9] Toma A, O'Neil D, Joffe M (2021). Quality of histopathological reporting in breast cancer: results from four South African breast units. JCO Glob Oncol.

[10] Spitaleri G, Toesca A, Botteri E (2013). Breast phyllodes tumor: a review of literature and a single center retrospective series analysis. Crit Rev Oncol Hematol.

[11] Chng TW, Gudi M, Lim SH, Li H, Tan PH (2018). Validation of the Singapore nomogram for outcome prediction in breast phyllodes tumours in a large patient cohort. J Clin Pathol.

[12] Usman A, Dahiru A, Baguda US, Adam S, Abdulmajid UF (2020). Metastatic recurrent malignant phyllodes in 17-year-old female. Ann Trop Pathol.

[13] Islam S, Maughn A, Bheem V, Harnarayan P, Naraynsingh V (2020). World's oldest case of synchronous bilateral benign phyllodes tumors of the breast: a rare occurrence. Cureus.

[14] Hashmi AA, Faraz M, Nauman Z (2018). Clinicopathologic features and prognostic grouping of gastrointestinal stromal tumors (GISTs) in Pakistani patients: an institutional perspective. BMC Res Notes.

[15] Hassan WA, Ibrahim R (2020). Expression of CD117, CD34, and VEGF proteins in progression from endometrial hyperplasia to endometrioid carcinoma. Int J Clin Exp Pathol.

[16] Hashmi AA, Iftikhar SN, Haider R, Haider R, Irfan M, Ali J (2020). Solid papillary carcinoma of breast: clinicopathologic comparison with conventional ductal carcinoma of breast. Cureus.

[17] Hashmi AA, Iftikhar SN, Munawar S, Shah A, Irfan M, Ali J (2020). Encapsulated papillary carcinoma of breast: clinicopathological features and prognostic parameters. Cureus.

[18] Hashmi AA, Munawar S, Rehman N (2021). Invasive papillary carcinoma of the breast: clinicopathological features and hormone receptor profile. Cureus.

[19] Hashmi AA, Zia S, Yaqeen SR (2021). Mucinous breast carcinoma: clinicopathological comparison with invasive ductal carcinoma. Cureus.

[20] Hashmi AA, Faraz M, Rafique S, Adil H, Imran A (2020). Spectrum of papillary breast lesions according to World Health Organization classification of papillary neoplasms of breast. Cureus.

[21] Naik N, Madani A, Esteva A (2020). Deep learning-enabled breast cancer hormonal receptor status determination from base-level H&E stains. Nat Commun.

[22] Bhattarai S, Kapila K, Verma K (2000). Phyllodes tumor of the breast. A cytohistologic study of 80 cases. Acta Cytol.

[23] Norris HJ, Taylor HB (1967). Relationship of histologic features to behavior of cystosarcoma phyllodes. Analysis of ninety-four cases. Cancer.

[24] Tsang JYS, Hui YK, Lee MA (2018). Association of clinicopathological features and prognosis of TERT alterations in phyllodes tumor of breast. Sci Rep.

[25] Atalay C, Kınaş V, Çelebioğlu S (2014). Analysis of patients with phyllodes tumor of the breast. Ulus Cerrahi Derg.

[26] Moten AS, Goldberg AJ (2019). Malignant phyllodes tumors of the breast: association between race, clinical features, and outcomes. J Surg Res.

[27] Chen CM, Chen CJ, Chang CL, Shyu JS, Hsieh HF, Harn HJ (2000). CD34, CD117, and actin expression in phyllodes tumor of the breast. J Surg Res.

[28] Sawyer EJ, Poulsom R, Hunt FT (2003). Malignant phyllodes tumours show stromal overexpression of c-myc and c-kit. J Pathol.

[29] Tse GM, Putti TC, Lui PC (2004). Increased c-kit (CD117) expression in malignant mammary phyllodes tumors. Mod Pathol.

[30] Tsuura Y, Suzuki T, Honma K, Sano M (2002). Expression of c-kit protein in proliferative lesions of human breast: sexual difference and close association with phosphotyrosine status. J Cancer Res Clin Oncol.

[31] Chen YC, Liao JW, Chang SC, Hsu WL (2020). Expression frequency of c-kit isoforms and its prognostic potential in canine mammary tumours. Vet Comp Oncol.

